# Progress of research on methods of human resource allocation in operating room nursing

**DOI:** 10.3389/fpubh.2025.1539108

**Published:** 2025-05-30

**Authors:** Qiaoling Zhang, Bijun Yu, Yangle Ou, Xiuran Zhou, Shifang Zou, Hui Peng, Xiaoying Yan, Tiemei Shen

**Affiliations:** ^1^Department of Nursing, Guangdong Provincial People’s Hospital (Guangdong Academy of Medical Sciences), Southern Medical University, Guangzhou, China; ^2^Department of Nursing, Guangdong Pharmaceutical University, Guangzhou, China

**Keywords:** operating theatre, human resource allocation, nurses, nursing management, manpower allocation methodology

## Abstract

The operating room is one of the most critical departments in a hospital, and the allocation of human resources for operating room nursing has a critical impact on operating room operations and surgical quality. This review systematically reviews current research on the content and methods of operating room nursing human resource allocation, including the quantity allocation, structure and quality allocation of operating room nursing human resources, and describes the specific operation, advantages and disadvantages of each allocation method, to provide a reference basis for the construction of a rationalized operating room nursing human resource allocation system.

## 1 Introduction

With the continuous development of medical technology and the increasing number of types of surgery, managers have increasingly emphasized the issue of human resource allocation in the operating room to ensure the quality of surgery and improve work efficiency. There is a mismatch between the number of nursing staff and the number of surgeries in many hospitals’ operating room nursing human resource allocation (1). The lack of nursing human resources and their irrational structure have led to increased workloads, longer shifts, and fewer training and learning opportunities for nurses, leading to a higher level of nursing risk (2). Due to the special nature of work in the operating room, current research on nursing human resource allocation often excludes operating rooms, ICUs, outpatient clinics, and other departments as platform departments from regular human resource allocation (3). Human resources allocation methods based solely on bed-to-nurse ratios and clinical standards of care are not directly applicable to the allocation of nursing human resources in the operating room (4). How to rationally allocate nursing human resources in the operating room to improve the efficiency and quality of surgery in the operating room has become an urgent problem that needs to be solved. This paper intends to review the current research on nursing human resource allocation in the operating room, including the quantity allocation, structure and quality allocation of nursing human resources in the operating room, and to summarize the methods and advantages and disadvantages of nursing human resource allocation in the operating room at home and abroad, to provide reference for operating room managers to allocate nursing human resources rationally.

### 1.1 Background

Herbert G. Hahnemann defines staffing as acquiring, placing, and retaining an adequate quantity and quality workforce to impact an organization’s effectiveness positively (5). Based on the in-depth study of this concept in business administration, human resource allocation can be categorized into micro and macro levels. The micro level mainly includes quantitative and qualitative allocation, while the macro level mainly refers to structural allocation (6).

While standards vary from country to country, a lack of human resources is a common problem. According to the implementation rules of China’s tertiary general hospital accreditation standards, the ratio of nursing staff to operating rooms under normal working conditions is ≥3:1 (excluding non-nursing staff such as auxiliary and administrative staff) (7). Some large and complex surgeries require more than three nurses of different seniority, e.g., lung transplantation involves four nurses in different roles (8). Lei ZY et al. (9) relied on various workload indicators from the operating room information management system to assess operating room nursing human needs and allocations. They found that the operating room nursing human resource allocation obtained according to the minimum ratio required by the national standard (3:1 ratio of nursing staff to interoperative rooms) was grossly inadequate.

Improving the efficiency of human resources is a problem that many managers have to solve. The work habits of the surgical team influence their solutions. Surgical teams in Chinese hospitals consist of fewer members. There is a low personnel turnover during this surgical procedure; scrub nurses or circulation nurses are stationary, and it is mainly the surgeon who undergoes turnover. In contrast, in operating rooms in Western countries, anesthesiologists and surgeons are fixed throughout the entire surgical procedure, and nurses are often transferred to other positions for various reasons (e.g., lunch and tea breaks), with frequent shift handovers occurring (10). One researcher found that surgical durations of more than 4 hours were associated with as many as six team member changes, most of which were nurses (11).

This difference has led to differences in the focus of researchers on staffing. Some Asian countries’ studies on human resource allocation in operating room nursing focus on optimizing scheduling, while Western countries have explored the turnover rate of intraoperative nurses, and how to staff the surgical process.

**Figure 1:**
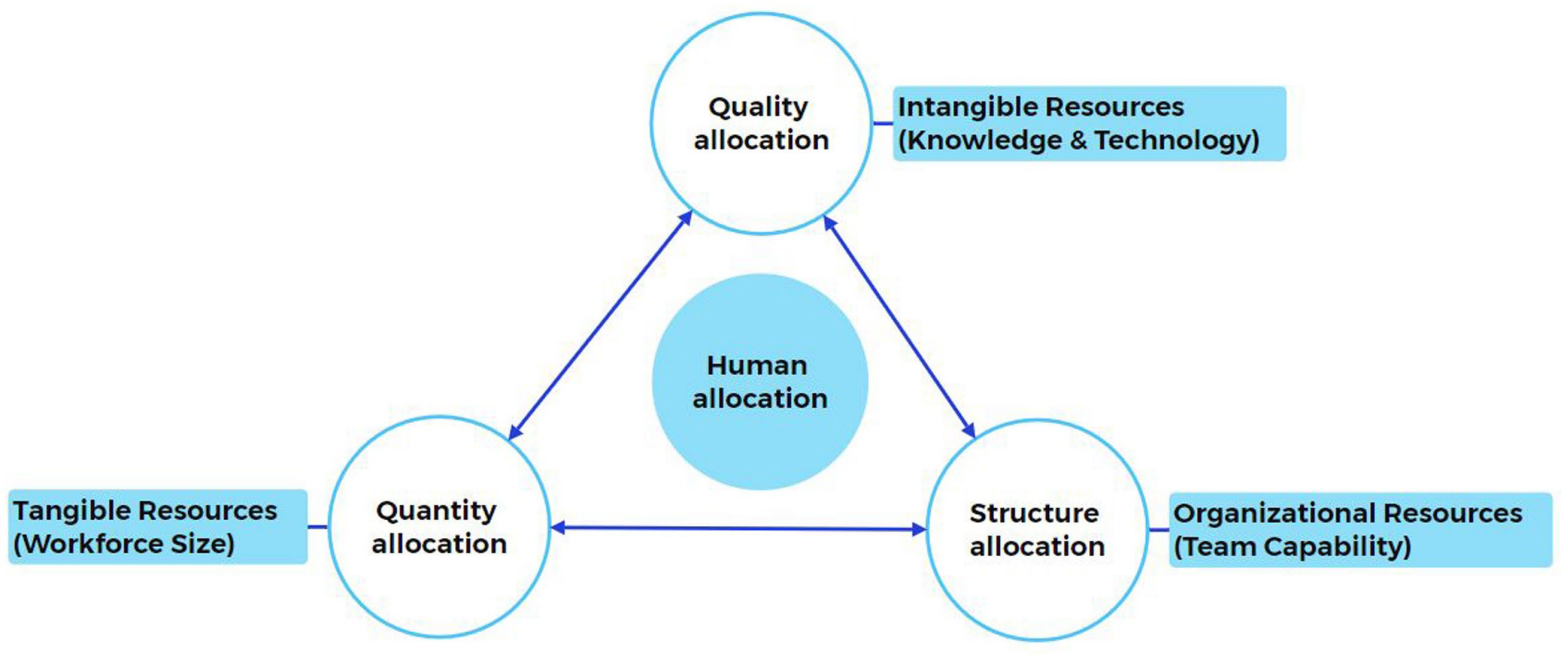
Human allocation model.

As illustrated in Figure 1, allocating OR nursing human resources requires a synergistic integration of three dimensions: quantity precision, structural rationality, and quality sustainability.

## 2 Results

### 2.1 Nursing human quantity allocation

#### 2.1.1 Projected human based on hours worked

The method of measuring working hours refers to measuring the time consumed by the procedures and actions that must be carried out in each part of the whole process of completing the work of a health service. It is the most basic method of determining the amount of labor (12).

As a classic method of nursing labor assessment, manpower requirement quantification is achieved by systematically recording the time spent on the whole process of nursing operations. Wang ZM’s team (13) obtained the total daily nursing work hours of nurses by measuring the work hours and frequency of each nursing program in the operating room, which was substituted into the human resource allocation formula for the calculation of nursing human resource allocation, which was calculated as follows: theoretical number of nurses per day = (total nursing hours per day/8 + 1) x rest factor x (1 + mobility factor). Where “8” refers to the daily working hours of nurses of 8 h; “1” refers to the workload of nurses on the main shift not counted at the time of measurement of working hours; rest coefficient = 365/(365 - rest days), the number of rest days mainly refers to legal holidays and weekly rest; mobility coefficient = the number of all vacation days in the year / (overall number of study participants × the number of whole days of attendance in the year), which refers to the number of supplements for nurses who are understaffed due to sick leave, maternity leave, personal leave and other leave of absence situations, as well as clinical teaching, and so on. Although this method can accurately reflect the clinical reality, its mode of relying on manual recording faces an efficiency bottleneck in contemporary medical scenarios with diversified surgeries and complex operation processes. In comparison, Lei ZY’s team (9) calculated the theoretical nursing human resource requirement by measuring the actual working hours of nurses each year based on the Ministry of Health’s proposed ratio of nursing staff to operating rooms of 3:1, which was calculated as follows: annual theoretical nurse requirement = [operating rooms × 3 + (overtime working hours per person per month × 12)/(regular working hours per day × actual number of working days)] × (1 + mobility factor). Although it has the advantage of simplicity of operation, the calculation is increased by the fact that it does not effectively differentiate between the time spent on nursing services and the time spent on non-nursing activities (e.g., tea breaks, administrative matters), which results in more hours than the actual hours spent on nursing services.

With the deep penetration of IoT technology in healthcare management, traditional work hour measurement methods are undergoing a technological revolution. Clinical practice has shown that the hospital base time distribution system constructed by a tertiary hospital in China can automatically collect the precise start and end times of nursing operations by deploying an NTP (Network Time Protocol) system and installing high-precision digital sub-clocks (time error ≤ 0.1 ms) in key units such as operating rooms and ICUs (14). A medical device monitoring system developed at the University Hospital of Heidelberg, Germany, on the other hand, has reduced the rate of nursing person-hour deflation from 12.7 to 4.3% by capturing real-time data on personnel movements in the operating room (number of personnel, time consumed for operation items, etc.) and generating a dynamic human resources demand curve in a visualization dashboard (15). The above technological breakthrough confirms that automated data collection based on IoT can significantly improve measurement validity and provide a reliable basis for dynamic human resources allocation.

Existing studies have generally ignored the moderating effect of surgical complexity in their analyses of the association between surgical duration and human resources requirements. Pandit’s (16) team proposed a model of time-phased human resources allocation (8-h/10-h team division criteria) based on the formula: workload = hours worked + K x overtime hours, where the relative cost ratio K value is > 1, and the section took the value of K to be 2.0. The following decision rule was calculated to be used for staffing anesthesiology and operating room nurses: 8 h of human resources were planned for staffing if the average length of actual operating room time used was ≤8 h and 25 min, and 10 h of human resources were planned for staffing if the average length was ≥8 h and 50 min. This method does not incorporate the differential impact of surgical difficulty, although it does consider the need for staff turnover during prolonged surgical procedures. To address this limitation, Chinese researchers constructed a system of difficulty coefficients for surgical coordination through the expert consultation method, integrating the four dimensions of surgical time, risk level, technical difficulty, and labor intensity to form quantitative assessment standards (e.g., appendectomy = 1.0, liver transplantation = 3.0), and incorporated them into the performance appraisal system. Post-implementation data showed that nurses’ annual per capita surgical coordination improved by 6.73%, and surgical pick-up time was reduced by 10.82% (17). The results have methodological consistency with the American RBRVS (Resource-Based Relative Value Scale) theory (18), which scores the coefficients of different medical projects through the four-dimensional indexes of time, skill, risk, and physical strength, and then obtains the workloads of other medical projects, which provides an opportunity to establish the association model of pervasive surgical complexity and human resources allocation theoretical support.

#### 2.1.2 Projected human based on systematic categorization

Surgical grading management refers to the process by which medical institutions, to ensure ensuring surgical quality and safety, grade the surgeries carried out in their institutions according to the degree of surgical risk, degree of difficulty, degree of resource consumption, and ethical risk, and adopt corresponding management strategies for surgeries at different levels (19).

In a survey of elective orthopedic surgeries (20), the researchers categorized nursing staffing using the level of surgery and the type of anesthesia as the main variables: those with a duration of surgery of less than 1 hour were not assigned a scrub nurse; those with a duration of surgery of more than 5 hours were assigned a circulation nurse, a scrub nurse, and an auxiliary staff member; and those who were in the middle of the two were assigned a circulation nurse and a scrub nurse. Except for general anesthesia patients with nurse anesthetists, patients with local or regional anesthesia, etc., no longer have nurse anesthetists. Compared to the traditional operating room (where a circulation nurse and a scrub nurse are assigned to each operating room), the efficient operating room is primarily due to the simplification of operating room care and elimination of some of the need for nurse anesthetists, with the result being a 60% reduction in the cost of procedures associated with the efficient operating room and equivalent health outcomes for patients.

American researchers graded patients according to general surgical anesthesia class (ASA) and surgical complexity (21), the sum was the patient’s classification number. For example, if a patient undergoing hepatectomy has an ASA score of 4 and a surgical complexity score of 2, the grade of the procedure is 6. The average number of nurses required per day was determined based on the current month’s data, and the procedure’s grading value determines the number of nurses needed in the operating room. Compared to the dynamic grading model for routine surgery, complex specialty surgery often requires rigid allocation criteria to ensure healthcare equity due to the higher ethical risks and resource consumption. In China, in the context of the universal health insurance policy, rigid allocation standards (2 circulation nurses and 2 scrub nurses) are implemented for major surgeries such as organ transplantation to ensure the stability of the surgical team (22). In contrast, the proportion of commercially insured patients in organ transplantation in the United States rose from 26.9 to 33.8% with the increase in the number of nurses, with a corresponding decrease in the proportion of patients with general health insurance (23).

In most hospital performance accounting systems, surgical grading accounts for a portion of the weight, but the dynamization of grading standards should be considered. In recent years, da Vinci surgery has been involved in all types of surgical procedures, in the face of new types of surgery, the specialized nurses and new types of surgery fit; in addition to the different tiers of hospitals, should also be taken into account, a study investigated 2,039 hospitals in China showed that the allocation of human resources for surgical care in tertiary hospitals is greater than the national standard of the ratio of nursing staff to the surgical interventions of 3:1, the second-level hospitals to meet the basic, and the majority of the first-level hospitals are not satisfied (24).

#### 2.1.3 Build a human resources model based on cost accounting

As the pressure to control costs in healthcare intensifies, cost accounting for nursing labor allocation in the operating room has become an important tool for hospital lean management. Eddie Y. Lo (25) and others optimized the allocation of human resources in the operating room based on cost accounting and obtained a “two-room” model for the shoulder surgery team (two operating rooms operating simultaneously, each staffed by one surgeon, one anesthesiologist, two nurses, and three ambulatory staff: one nurse, two assistants, and two assistants). A cost comparison of the optimized team model with the traditional “one-room” surgical team model (1 surgeon, 1 anesthesiologist, 2 nurses, and 1 assisting staff in each operating room) resulted in a 23% reduction in operative time and $187,000 in annual costs. The model is innovative in incorporating space reuse and staff mobility efficiencies into the costing framework, but its success relies on rigorous surgical timing synergies and equipment-sharing mechanisms.

Time-Driven Activity-Based Costing (TDABC) is often used for healthcare costing. A study in the United States (26) measured the time of each nursing operation throughout the surgical procedure based on the time-driven operations cost theory (TDABC). It calculated the cost required for each nursing time phase, using a surgical flow chart and the corresponding time values to determine the total labor costs associated with the degree of activeness (use) and idleness (un-usedness) of the other personnel in each nursing phase during the surgical procedure. TDABC allows for a detailed analysis of the labor costs of all parties in a surgical team, and researchers have generated several new labor allocation models based on different team roles to explore the most cost-effective and efficient models for the team. Costing based on TDABC theory not only provides a detailed understanding of the total labor cost of the team but also provides a detailed understanding of the time usage of each team member. Alternative staffing models can also be calculated to maximize labor cost savings and improve team efficiency. However, the static assumptions of traditional TDABC (fixing the duration of surgery and ignoring emergencies) lead to its limited applicability in complex surgeries, such as heart transplantation, where the actual cost deviation rate is 21.3% (26).

Although the current operating room human resources allocation model based on cost accounting has made a breakthrough at the theoretical level, its clinical application is still faced with a triple dilemma: (1) Conflict between cost control and quality of care (e.g., the “two-room” model reduces the cost of an operation by $291, but nurses running back and forth between the two operating rooms will also increase the error rate of nursing operations (26)); (2) A contradiction between static accounting and dynamic demand (traditional TDABC does not include the time cost of intraoperative emergencies); (3) An imbalance between economic considerations and humanistic care (excessive compression of human resources increases nurse fatigue index (27)). The practice of the German G-DRG system has shown that coupling the case-mix index (CMI) with the nursing efficiency factor (NEF) can increase the costing accuracy to 88%. However, the model must be matched with a mature DRG payment system (28).

Based on this, some scholars have proposed an integrated program: under the DRG framework, TDABC is used to account for indirect nursing costs (e.g., depreciation of equipment, space occupation) and combined with the RBRVS theory to quantify the direct nursing labor costs (weighted according to risk, skill, and physical exertion), and then obtain a more accurate cost of care. This method takes into account not only the number of patients admitted to the emergency comprehensive care but also the number of patients with a variety of diseases. Care is not complex, but the burden of care is heavy. However, it also takes into account the critical care unit, such as the nursing unit of nursing experience and skill requirements of the characteristics of the higher, but also to achieve the set up of virtual beds nursing unit of the measurement of the performance point, reflecting the characteristics of the care of the different departments, to achieve to the maximum extent possible to do the relative balance of the configuration of the performance point (29).

#### 2.1.4 Mathematical modeling of human resources numbers

The application of computer technology allows for the simultaneous programming of human resource requirements, human resource allocation, and departmental scheduling. Some studies (30) proposed a two-stage mathematical model for long-term employee planning decisions in the operating room. The first stage is a stochastic model that calculates the annual demand for nurses in the department based on the hospital’s budgeted cost planning, which results in the expected weekly hours of work for all nurses and sets the conditions for employees to work overtime in order to meet the changes in demand. The second stage of decision-making focuses on determining the staffing structure, i.e., the number of staff assigned to each shift, each workday, and each service line, considering the number of nurses available and the pattern of demand. The advantages of this method are that once the programming is successfully implemented in the clinic, it saves human and material resources, is easy to operate and easy to manage, and can be personalized according to the specific conditions of the department to cope with the day-to-day problems of departmental human resources and surgical scheduling. However, it may not be able to achieve complete improvisation in response to unexpected situations in the department. Most current mathematical models of the operating room are mostly seen in the context of surgical scheduling.

The current application of mathematical modeling in operating room human resources deployment presents a significant contradiction between “technological sophistication and clinical appropriateness.” HeBei of hospital’s digital twin scheduling system achieves a 38.07% increase in burst response efficiency through Nash equilibrium. However, its training model relies on historical data is still subject to model prediction errors in rapidly iterative scenarios such as the annual growth of da Vinci surgeries (31). The current core problem lies in the following: the existing models rely excessively on static assumptions, such as fixed operation duration, ignoring the dynamic complexity of medical scenarios, and over-pursuing mathematical optimal solutions, weakening the quantitative expression of humanistic elements. In order to break through this double dilemma, there is an urgent need to construct an intelligent modeling system that integrates dynamic perception and humanistic constraints. A multimodal data perception model is established, and a single data model is fused with real-time surgical video (identifying the frequency of instrument delivery) and vital signs data (calculating the intensity of nursing operation) to construct dynamic feature vectors.

To clarify the characteristics of different staffing models, a comparative summary is provided in Table 1, highlighting their applicability and limitations in diverse clinical contexts.

**Table 1:** Characteristics of the method of nursing human quantity allocation.

Model type	Core metrics	Advantages	Limitations	Case examples
Work hours measurement	Work hour tracking, Rest coefficient, Mobility factor	Accurately reflects clinical realities (13)	Relies on manual recording, low efficiency (9)	NTP system in Chinese tertiary hospitals (14)
Surgical categorization	Procedure classification (Risk/Difficulty/Ethical level)	Differentiated allocation, improves equity (21)	Static classification fails to adapt to new technologies (24)	ASA classification in the US (21)
TDABC Cost model	Time-driven costs, Indirect/Direct care costs	Precise team cost accounting (26)	Ignores emergency time costs (26)	G-DRG system in Germany (28)
Mathematical modeling	Dynamic scheduling algorithms, Historical data training	Addresses surge demands (30)	Over-reliance on static assumptions (31)	Digital twin system in Hebei Hospital (31)

### 2.2 Nursing human structure allocation

In addition to considering the number of personnel in the job setup, it is also necessary to ensure that the structure and distribution ratio of nursing personnel at all levels can be reasonably configured to ensure the quality of nursing care, stimulate the potential of nurses, and improve the efficiency of the role’s work.

Based on their core competencies, nurses are most often categorized in Western countries as clinical nurse specialists, specialist nurses, advanced practice nurses, and licensed practical nurses (32). The Chinese nursing team categorized OR nurses into N1 (Junior Responsible Nurse), N2 (Responsible Nurse), N3 (Senior Responsible Nurse), and N4 (Responsible Team Leader) or grading OR nurses based on years of experience and appointment to different job tiers of OR nurses (24, 33), nurses are assigned to the appropriate surgical care based on their rank. Wang SQ et al. (34) constructed an evaluation system for the comprehensive competence of nurses’ job competence based on their professional knowledge, nursing skills, communication and coordination skills, organizational and management skills, professionalism, personal characteristics, and career planning. In order to provide nurses with a more suitable working environment and development opportunities and, simultaneously, provide patients with more comprehensive, professional, and humanized nursing services.

Some hospitals (35) through a comprehensive assessment of the riskiness, complexity, professional skill requirements, and workload elements of nursing work programs. The operating room nursing work program was divided into four tiers, and based on the requirements for practitioners in the four tiers, the criteria for tiering operating room nursing positions were determined, and recommendations for tiering the use of nursing staff were made. That is, through the nursing positions in the operating room for job analysis, to determine the various levels to take different qualifications of nurses to do the corresponding level of nursing work projects and to serve as the corresponding level of responsibility to allocate the nursing human resources in the operating room rationally.

The traditional model of pairing circulating nurses with scrub nurses is the new-old nurse pairing model, nursing managers (6) and others analyzed the psychological traits of operating room nurses and found that complementary matching of nurses with different psychological traits forms a complementary matching model of psychological traits conducive to improving the business quality and work enthusiasm of specialized nursing staff in the operating room, strengthening the concept of nurse service, and thus optimizing the allocation of nursing human resources in the operating room. However, putting aside conditions such as nurse seniority and education, using psychological traits alone as the basis for surgical nursing pairings may result in two nurses with insufficient seniority and experience appearing in the same surgery, reducing the degree of cooperation between the nursing team and the anesthesia and surgeon teams.

The essence of nursing human resources configuration in the operating room is to realize the dynamic adaptation of “doctors, nurses, and patients,” and the core challenge is how to accurately match nurses’ explicit competencies (e.g., level of specialty certification, the precision of microscopic operations) and implicit characteristics (e.g., the tendency for teamwork, stress response mode) with the technical demands of the type of surgery (e.g., the cognitive load of the instruments for robotic surgeries), and the operating habits of the operating surgeons (e.g., preferences for the pace of surgery, team communication styles) and team communication style for precise matching. Different hospitals have different requirements for the above-influencing factors. Specialized oncology hospitals consider the difficulty coefficient of surgical oncology coordination, patient criticality, knowledge and skills, title, nurse hierarchy, and interpersonal interactions with colleagues in the allocation of human resources structure (36). It is a bit more simplified in primary hospitals, and staff will be deployed based on a single nurse tier and surgical grading (37). Establishing a comprehensive nursing human resources structure allocation system based on the nature of the healthcare organization can maximize the matching of people and positions.

### 2.3 Nursing human quality allocation

In business administration, human resource allocation quality refers to assigning personnel to the corresponding job positions according to their responsibilities. To maximize the role of human resource quality allocation, enterprises should achieve the goal of matching employees with positions, employees with each other, and employees with enterprises (6).

#### 2.3.1 Reasonable qualitative allocation of human resources can compensate for a certain degree of quantitative human resources shortfalls

From the engineering perspective, the shortage of human resources and the lack of strong professional knowledge of employees can lead to problems in the scheduling of personnel and impede the optimal allocation of human resources, thus affecting the overall quality of work and the direction of the goals of the enterprise. The problems caused by a small number of vacancies in the number of employees can be compensated to a certain extent through reasonable staff scheduling and rostering, thus ensuring the effective use of human resources (38).

##### 2.3.1.1 Joint specialized team on flexible scheduling system

Human resource allocation for operating room nursing in China uses a combination of cascade allocation and flexible scheduling. According to the professional skills, work experience, education, and title level of the nurses, the nursing staff were divided into three levels, namely, specialty team leader, senior responsible nurse, and junior responsible nurse, to ensure that there were nurses of different levels on duty in all shifts to ensure the quality of nursing care under different surgical needs (39). Some hospitals specialize managing operating room nurses by dividing them into specialty groups. Through core competency assessment and grading, we ensure that the specialty teams have specialized nursing competencies to meet the needs of complex surgery and emergency resuscitation. In the United States, specialty surgical teams are used to optimize operating room workflow, an approach in which clinicians lead the formation of a multidisciplinary group of teams, with team members (including operating room nurses and anesthesiologists) sharing common goals and a process of open discussion about the needs of each team member (40). This approach allows for more specialized and proceduralized care in the operating room. Further, it improves the ability of nurses to provide standardized and efficient care and coordinate specialized procedures.

The nurse manager schedules shifts based on surgeon visits, consultations, procedures, and nurse tiers during the week. At the same time, flexible scheduling is carried out according to the patient’s medical needs and based on guaranteeing nurses 2 days of rest per week, avoiding overloading of nurses and reducing the risk of nursing errors and doctor-patient disputes (41). A combination of hierarchical management and flexible scheduling has been adopted to flexibly adjust the working hours of nurses according to the length of surgery and workload, reduce the number of shift handovers, and improve work efficiency.

##### 2.3.1.2 Emergency response mechanisms and mobile nurse pools

The common problem faced by nursing human resources in many countries at present is the shortage of nurses’ resources, especially during the peak flow hours of surgery, and many researchers have proposed corresponding countermeasures on how to supplement the overall shortage of human resources in the operating room. On the one hand, hospital management should establish an emergency response mechanism, set up a special nursing management team for emergencies and special situations, scientifically assess nursing human resources, and formulate the hospital emergency coordination and departmental emergency plans (42, 43). On the other hand, hospitals have established “nurse banks” to make up for the shortage of nurses or as a potentially effective way of responding to changes in demand (44), but studies have shown that the unfamiliarity of these nurses with the operations of the operating room unit since they come from an external agency nurse or in-house nurse has led to an increase in the number of adverse events in nursing care (45); Drawing on overseas “nurse banks,” some hospitals in Asia have set up “mobile nurse banks” and “hourly nurse banks” to cope with seasonal or daytime peak hour nursing work demands (46), but how to localize foreign theories is still in the exploratory stage. There is a lack of a complete scientific and mature system (46).

Reasonable quality allocation of human resources can partially offset the shortcomings of insufficient quantity by improving individual effectiveness and teamwork efficiency. However, the boundaries of its role and the path to its realization need to be scientifically defined (6). Australian hospitals have collected information on surgeries and nurses and constructed a prediction model of daily surgical caseload, with a prediction accuracy of more than 90% and consistent prediction performance over various prediction ranges, so that this method can provide information for short-term staffing choices and long-term strategic planning (47). Tertiary care hospitals can implement a “1 + N” expert-led model (1 specialist nurse leader guiding N nurses), which dynamically adjusts skill weights in conjunction with the demands of new technologies such as the da Vinci surgical robot. Primary hospitals should adopt the “general nurse + remote consultation” model, connecting nursing experts in higher hospitals in real-time through 5G operating rooms to make up for the shortcomings of specialized capabilities (48).

#### 2.3.2 Optimizing the allocation of human resources structures improves quality allocation

Turning to nursing administration, the definition of post-competency (PC) exhibits core concepts similar to business administration’s. It refers to the objectively measurable knowledge, skills, attitudes, values, personality traits, and motivation of high performers in a particular job, organizational environment, and culture (49). It is worth discussing how to realize the reasonable allocation and optimization of the competency of nurses in the operating room, which has become an important issue that needs to be analyzed and solved urgently. There are currently two main models of post-competency: the iceberg model and the onion model. According to the principle of the iceberg model, post-competencies can also be divided into explicit (including knowledge, skills, and resources) and implicit (including self-worth orientation, potential plasticity, and personality traits) competencies (50); The onion model, on the other hand, refers to the fact that post competencies are distributed like an onion, from the inside out: motivation, personal characteristics, self-image, social roles, knowledge, and skills (51). The Association of Operating Room Nurses (AORN) defines the competencies of an OR nurse as the knowledge, skills, and abilities needed to perform the professional functions of a registered nurse in operating room (52).

Several studies (53, 54) have demonstrated that post-competency of nurses can be improved through specialized training methods. In the case of surgical nurses, Some researchers (54) improved the post-competence of nurses by training midwifery nurses in *In Situ* Simulation (ISS), a simulation teaching strategy based on experiential learning theories, situational cognition theories, and constructivism, among other theories. It involves bringing the simulation suite to a real healthcare unit and using resources such as instruments and equipment from that unit while requiring participants to simulate their training by their real-life job roles, skill levels, and areas of responsibility.

The current post-competency management system for operating room nurses faces a significant disconnect between theory and practice, and there is an urgent need to build a dynamic assessment and training system that meets actual clinical needs (52). Although traditional competency models (such as the iceberg and onion models) provide a basic framework for the division of competencies, they fail to effectively address the complex interaction of competencies in the operating room scenario. For example, in da Vinci robotic surgery, nurses need to simultaneously possess three-dimensional spatial cognition of instruments (explicit skills), team coordination under stress (implicit traits), and the ability to learn new technologies (potential plasticity) quickly. Quantifying the composite requirements of such multidimensional competencies in the existing models is difficult. Improvements need to focus on building a dynamic competency model: constructing a three-dimensional matrix (technical operation/teamwork/innovative adaptation) and dynamically adjusting the weights according to the type of surgery (e.g., focusing on nurses’ innovative and adaptive abilities in robotic surgery).

#### 2.3.3 Innovative techniques to optimize the overall quality of human resources allocation in the department

As the concept and technology of Artificial intelligence (AI) began to be applied medical care and management, scholars at home and abroad have gradually explored and optimized it. Some scholars monitor the workload and staffing situation in the operating room in real-time by establishing a nursing human resource management system to provide data support for dynamic adjustment. One study (55) used queuing theory to calculate the allocation of nursing human resources in the operating room when peak surgical hours occur. Some researchers (56) designed a robot that utilizes a speech recognition system and computer vision technology to assist human scrub nurses in locating and transferring instruments. Equipped with an electromagnetic gripper that recognizes 27 instruments and responds to 82 spoken commands, the robot reduces the workload of scrub nurses and addresses surgical staffing shortages.

The current application of AI technology in operating room human resources allocation has the dual limitations of “focusing on technology rather than practicality” and “disconnecting from the actual situation.” Lim’s queuing model can theoretically deduce the human resources demand during peak hours, but it fails to incorporate the surgical emergencies and nurses’ skill differences, which may lead to prediction bias. Rekha’s instrumentation robot reduces some of the physical load on scrub nurses. However, its 82-command set does not cover the changing needs of complex surgical instrumentation, and electromagnetic grasping accuracy errors may pose a risk of instrument injury (56). In the process of improvement, it should be noted that when calculating the human resources requirement, the difficulty of surgery and the level of nurses should be taken into account so that the prediction can be more accurate; VR simulation of human-machine cooperation scenarios should be used to cultivate robotic nurses, so that nurses can practice handling emergencies in advance, and the operation errors can be reduced.

## 3 Discussion

### 3.1 Artificial intelligence-driven mechanism for dynamic human resources deployment in the operating room

In exploring the process of human resource allocation in operating room nursing, a core element that cannot be ignored is the flexibility of allocation and the close integration of the actual situation. From business administration to nursing management, human resource allocation has always emphasized the importance of matching people to jobs, people to people, and companies, which is also applicable and crucial in operating room nursing (38). As a high-intensity, high-risk working environment in hospitals, the operating room is highly required for nursing staff’s professional skills, work experience, and adaptability. Therefore, the special characteristics and complexity of the operating room must be fully considered when allocating human resources. However, flexible deployment does not mean arbitrary arrangement. In the allocation process, the nurses’ professional skills, work experience, and physical and mental conditions must be fully considered to ensure that they can play the greatest role in the positions they are capable of. Therefore, allocating nursing human resources in the operating room must be closely integrated with the actual situation to achieve flexible and efficient deployment.

With the deep integration of AI and IT technologies, the future needs to focus on developing intelligent scheduling systems with multimodal data fusion. For example, a dynamic prediction model can be constructed by integrating multidimensional parameters such as discrete coefficients of surgical duration, distribution of anesthesia types, and nurse competency mapping. At the same time, it is necessary to design specifications for human-robot collaboration, such as setting the operating error threshold (≤0.2 mm) and emergency takeover conditions (bleeding >300 mL) for robotic nurses and realizing the traceability of the whole process of operation through blockchain technology to ensure clear attribution of responsibility.

### 3.2 Interdisciplinary optimization of nurses’ career development paths based on person-position matching

Operating room nurses generally suffer from a lack of staffing, work overload, few promotion opportunities, and high promotion requirements. These problems not only affect nurses’ work motivation and career satisfaction but also directly affect the quality of nursing care and patient safety in the operating room (8). By establishing a reasonable incentive mechanism, nurses’ work motivation and creativity can be stimulated. Optimize the compensation standards and programs for nursing staff, maintain the stability of the nursing team, and stimulate the vitality of nursing staff.

Encourage nurses to develop in many ways and train multidisciplinary nursing personnel. There are still some professional barriers between nursing and other professions. For example, at present, the allocation of nursing human resources in the operating room mainly based on the work experience of the head nurse in the department, and the cross-study between nursing and business administration can make the allocation of human resources in the operating room more scientific; the adjustment of the hospital information system will bring about changes in the work of nurses, and the optimization of the hospital information system can reduce the problem of nurses repeatedly writing nursing documents and so on. One study developed a nursing shift handover information system based on an electronic medical record system based on the formation of a multidisciplinary team and the development of a structured nursing shift handover form, which led to a reduction in the length of nurses’ shift handover preparation by 14.05 min, an increase in the completeness rate of the elements of the shift handover record by 5.01%, and a decrease in the rate of errors in the information in the shift handover report by 8.31% (57). The system adjustment by the hospital information department can only be gradually adjusted in brief communication with the nurses. The final effect does not fully meet the needs of nursing. Culturing nursing and computer information technology talents can substantially improve the medical security capacity and efficiency of the entire hospital and solve the needs faced in the actual medical applications.

### 3.3 Synergistic development of innovative technology and quality and safety in care

The premise of nursing human resource allocation is to ensure nursing quality and safety. Future research needs to establish a quality-resource allocation linkage assessment system to realize the forward movement of quality control. For example, biosensors and computer vision systems are deployed at key surgical nodes to monitor real-time instrument count accuracy and team response timeliness, automatically triggering the mechanism of human resources replenishment. The Xi’an Honghui Hospital has significantly improved its minimally invasive surgery coordination ability by establishing an operating room nursing group, adopting the “theory + practice + scenario simulation” model, and reducing the intraoperative instrument assembly error rate by 41% (58). In addition, it is necessary to incorporate quality of care indicators (such as postoperative infection rate) into DRG payment weights, design elasticity adjustment models (such as the coefficient of care for level IV surgery × 1.8), and promote the transformation of health insurance payment from “payment according to quantity” to “payment according to quality” (17).

Considering that nurses in the operating room are taking on more specialized work, human resource allocation should cover non-nursing time such as pre and post-surgical patient transfer, cleaning and disinfection, and so on. Nursing managers should create additional auxiliary staff to realize the specialization of professional staff, provide more rest time for nurses, reduce workload, and improve satisfaction and quality of care. In addition, the construction of intelligent and digital operating rooms is being promoted. By introducing medical information system technology, the intelligentization of surgical scheduling and resource allocation can be realized to solve the problem of nursing human resources allocation at different times in the operating room.

At the same time, it streamlines surgical preparation steps, improves equipment utilization efficiency, strengthens internal communication and collaboration, reduces waiting time, and enhances operational efficiency. Since the introduction of the first da Vinci robotic surgery in China in 2006, various types of intelligent surgical equipment have been emerging, and the care process has been optimized in a continuous manner (59). Wang HZ’s team (60) used AI technology to build a parallel healthcare system consisting of a real healthcare system and the corresponding one or more virtual or ideal artificial systems, and through online learning, offline computation, and interaction between the real and virtual worlds, the AI becomes an experimental “social laboratory” to achieve personalized training, medical decision-making, and behavioral assessment of healthcare workers, in order to achieve more accurate and scientific medical control. With the rapid development of AI, nursing human resource allocation will be more accurate in terms of quantity, structure and quality, which will contribute to better management of human resources in hospitals. Therefore, optimizing nursing human resource allocation in the operating room needs to comprehensively consider the construction of intelligence, non-nursing work time human resources demand, and sustainable team development in order to achieve efficient utilization and sustainable development and to improve the operational efficiency and quality of care.

### 3.4 Cross-cultural comparison of staffing priorities

While technological integration reshapes local practices, a cross-cultural comparison, as analyzed below, reveals more deeper systemic divergences in staffing priorities.

Stability versus flexibility from a team structure perspective. Chinese operating room nursing teams use a fixed hand-washing nurse model (9), especially in complex surgeries such as organ transplantation, where a rigid configuration of “2 hand-washing nurses + 2 traveling nurses” is implemented (22). The West is characterized by “mobile nurses + fixed doctors” (10), with up to 6 nurse rotations occurring in a single 4-h surgery (11), which improves efficiency but increases the risk of handover errors (27). This discrepancy reflects the divergence of cultural values that prioritize safety versus efficiency orientation.

Differences in administrative compliance and market regulation approaches in scheduling mechanisms. Asian countries ensure policy compliance through the 3:1 nurse-operating room ratio (24) and Japan’s “super nurse” extended scheduling system (59). In contrast, Europe and the United States use the TDABC model to quantify the cost of nurse idleness (26), and Germany’s G-DRG system optimizes human resources inputs through the CMI index (28). Both types of models face the challenges of inadequate dynamic demand response and excessive cost control, respectively.

Technology paths reflect centralized infrastructure and decentralized innovation governance. China relies on a government-led NTP time synchronization system for centralized data control (14), and 5G remote collaboration to bridge grassroots capacity gaps (60). Western hospitals autonomously choose the RBRVS performance model (18) and promote the standardization of da Vinci surgical instrument delivery (56), with differences in technology choices reflecting fundamental divergences in healthcare system governance models.

## 4 Outlook

Future research needs to break through the traditional experience management model through intelligent technology empowerment, quality and safety integration, and policy system construction to promote the operating room nursing human resource allocation from “static experience-based” to “dynamic, intelligent” leap. This requires not only technological innovation (e.g., digital twin system) but also multi-dimensional synergy of policy support (e.g., flexible DRG payment) and humanistic care (e.g., nurses’ career development paths) and ultimately realizes the triple enhancement of patients’ safety, nurses’ well-being, and hospitals’ benefits.

## References

[1] Zhu M Chen C Feng H Wang ZG Liu CH Jiang P. Research on surgical workload performance appraisal oriented by high-quality development evaluation in public hospitals. Chin Hospitals. (2023) 27:35–7. doi: 10.19660/j.issn.1671-0592.2023.06.09

[2] Biparva AJ Nikjoo RG Jannati A Arab M Ostadi A. Challenges and prerequisites of risk management program in the operating rooms of Iranian hospitals: a qualitative study. J Educ Health Promot. (2023) 12:407. doi: 10.4103/jehp.jehp_245_23, PMID: 38333151 PMC10852172

[3] Wei LL Li HY Song L Hu ZJ Xiu H Huang X. Nursing human resource allocation based on working state of nursing units. J. Nurs. (2018) 25:19–23. doi: 10.16460/j.issn1008-9969.2018.05.019

[4] Wang Y Wang H Zeng TY Liu Y Wang Y Li MQ. Research of nurse staffing policy in China based on a three-dimensional framework. J Nurs Sci. (2024) 39:77–80. doi: 10.3870/j.issn.1001-4152.2024.10.077

[5] He LJ Yan ZZ Liu L Huang XG. Survey on human resource allocation of the first - class general hospitals in Jiangsu. Med Soc. (2013) 26:41–3. doi: 10.3870/YXYSH.2013.11.014

[6] Li HJ. Study on the optimization of human resource allocation in B medical company China University of Petroleum (2023). Available at: https://kns.cnki.net/kcms2/article/abstract?v=vG2M3utQQCc8N1uq8cBS-AMZo3ROkD0UMp_x0U0pZaSFX5XnTbTIM_NI-SaxQVWwEB5wF_kStFOZNPlLZYNphDF5gIrT38AM3pb9-N5I97auxnx21idVb4_Prk1yf5MV3kuBMuuKPWtTXlFtiJBLhcYAILWdB2Fb9pQuBtdxG84=&uniplatform=NZKPT

[7] The Ministry of Health has issued medical quality management and control indicators for tertiary general hospitals. Available at: https://www.nhc.gov.cn/wjw/c100175/201101/be9f9c2646b848249a8cd32f023b2a11.shtml (Accessed December 4, 2023).

[8] Wang XJ Chen B Mei J Zheng PP Chen X Chen C. Expert consensus on lung transplantation nursing (version 2022). Chin J Clinic Thoracic Cardiovasc Surg. (2022) 29:1395–401. doi: 10.7507/1007-4848.202209044

[9] Lei ZY Huang DH. Survey and analysis of human resource allocation and demand in operating room of a tertiary hospital. Chongqing Med. (2015) 44:3851–3. doi: 10.3969/j.issn.1671-8348.2015.27.037

[10] Cassera MA Zheng B Martinec DV Dunst CM Swanström LL. Surgical time independently affected by surgical team size. Am J Surg. (2009) 198:216–22. doi: 10.1016/j.amjsurg.2008.10.016, PMID: 19285305

[11] Sykes M Gillespie BM Chaboyer W Kang E. Surgical team mapping: implications for staff allocation and coordination. AORN J. (2015) 101:238–48. doi: 10.1016/j.aorn.2014.03.018, PMID: 25645040

[12] Wang H Wu M Song YL Han ZF Fan KL Jiang Y. Research on “amoun to funit time services” and its application on regional health manpower allocation. Chinese Health Res. (2000) 6:283–5.

[13] Wang CM Yu XR Huang JZ Li C Sun LQ. A study of labor and delivery room midwifery human resource allocation based on work hour measurement. Nurs Rehab. (2023) 22:72–6. doi: 10.3969/j.issn.1671-9875.2023.04.018

[14] Yan Y Peng L Wang YQ Wen J. Construction and optimization of the hospital benchmark time release system. China Digital Med. (2025) 20:78–82. doi: 10.3969/j.issn.1673-7571.2025.03.011

[15] Klar O Klass M Schneider G Kenngott H Heinze O. Estimation and monitoring of operating room utilization by a distributed streaming and analytics architecture deployed at Heidelberg University Hospital’s medical data integration center. Stud Health Technol Inform. (2022) 290:345–9. doi: 10.3233/SHTI220093, PMID: 35673032

[16] Pandit JJ Dexter F. Lack of sensitivity of staffing for 8-hour sessions to standard deviation in daily actual hours of operating room time used for surgeons with long queues. Anesth Analg. (2009) 108:1910–5. doi: 10.1213/ane.0b013e31819fe7a4, PMID: 19448221

[17] Wei YT Wu XH Chen TH Teng Y. Construction and effect analysis of the performance appraisal system in the operation room. Chinese Nurs Manage. (2016) 16:1230–5. doi: 10.3969/j.issn.1672-1756.2016.09.018

[18] Hickey PA Gauvreau K Jenkins K Fawcett J Hayman L. Statewide and national impact of California’s staffing law on pediatric cardiac surgery outcomes. J Nurs Adm. (2011) 41:218–25. doi: 10.1097/NNA.0b013e3182171b2e 21519208

[19] Li XL. Notice of the general Office of the National Health Commission on printing and distributing the measures for the grading of surgical procedures in medical institutions. Available at: https://www.nhc.gov.cn/wjw/c100175/201101/be9f9c2646b848249a8cd32f023b2a11.shtml

[20] Chohan M Bihari A Tieszer C MacNevin M Churcher C Vandersluis C. Evaluation of a tiered operating room strategy at an academic Centre: comparing high-efficiency and conventional operating rooms. Can J Surg. (2022) 65:E739–48. doi: 10.1503/cjs.004021, PMID: 36347535 PMC9648662

[21] Bell L. Using OR patient classification for staffing assignments. AORN J. (2015) 101:639–49. doi: 10.1016/j.aorn.2015.03.003, PMID: 26025740

[22] Liu F Chen SY Yang LN Bai XX Liu B. Development of a novel mode of human resource allocation in the operation room for organ transplantation and its clinical practice research. Prac J Clinic Med. (2017) 14:74–7. doi: 10.3969/j.issn.1672-6170.2017.03.024

[23] Ross HI Jones MC Hendriksen BS Hollenbeak CS. Nurse staffing and outcomes for pulmonary lobectomy: cost and mortality trade-offs. Heart Lung. (2021) 50:206–12. doi: 10.1016/j.hrtlng.2020.12.001, PMID: 33302148

[24] Guo L Mi XQ Chen XM Xu M He L Chang HC. Nursing human resource management status of operating room from 2039 hospitals in China. Chinese Nurs Manage. (2017) 17:1014–9. doi: 10.3969/j.issn.1672-1756.2017.08.002

[25] Lo EY Bowler J Lines T Melton C Volkmer R Majekodunmi T. Operating room efficiency and cost reduction in shoulder arthroplasty: is there an advantage of a dedicated operating room team? JSES. (2021) 31:125–30. doi: 10.1053/j.sart.2020.11.002

[26] Balakrishnan K Goico B Arjmand EM. Applying cost accounting to operating room staffing in otolaryngology: time-driven activity-based costing and outpatient adenotonsillectomy. Otolaryngol Head Neck Surg. (2015) 152:684–90. doi: 10.1177/0194599814568273, PMID: 25623288

[27] Hara K Tachibana R Kumashiro R Ichihara K Uemura T Maeda H. Emotional analysis of operating room nurses in acute care hospitals in Japan: insights using ChatGPT. BMC Nurs. (2025) 24:30. doi: 10.1186/s12912-024-02655-9, PMID: 39789556 PMC11716517

[28] Baumgart DC le Claire M. The expenditures for academic inpatient Care of Inflammatory Bowel Disease Patients are Almost Double Compared with average academic gastroenterology and hepatology cases and not fully recovered by diagnosis-related group (DRG) proceeds. PLoS One. (2016) 11:e0147364. doi: 10.1371/journal.pone.0147364, PMID: 26784027 PMC4718463

[29] Shi C Chen JR Chen GH Xue SM. Study on design and application of the calculation model of nursing unit performance points in public hospitals. Health Econ. Res. (2023) 40:76–80. doi: 10.14055/j.cnki.33-1056/f.2023.03.012

[30] Villarreal MC Keskinocak P. Staff planning for operating rooms with different surgical services lines. Health Care Manag Sci. (2016) 19:144–69. doi: 10.1007/s10729-014-9307-x, PMID: 25366968

[31] Xue J Li Z Zhang S. Multi-resource constrained elective surgical scheduling with Nash equilibrium toward smart hospitals. Sci Rep. (2025) 15:3946. doi: 10.1038/s41598-025-87867-y 39890977 PMC11785977

[32] Leary A Maclaine K Trevatt P Radford M Punshon G. Variation in job titles within the nursing workforce. J Clin Nurs. (2017) 26:4945–50. doi: 10.1111/jocn.13985, PMID: 28880423

[33] Luo L Hu WZ Jiang Y Zheng XL Liu SS Leng HY. Research Progress on hospital nursing workforce capacity planning and control. Indus Eng Manage. (2023) 28:184–98. doi: 10.19495/j.cnki.1007-5429.2023.05.019

[34] Wang SQ Chen LF Qian YX Hou LH. Construction of a post competency evaluation index system for nurses in the hybrid operating room. J Jinan Univ. (2023) 44:289–96. doi: 10.11778/j.jdxb.20230053

[35] Pang SY. A study of stratification criteria for operating room nursing positions based on job analysis - a case study of a tertiary public hospital. Heilongjiang University of Chinese Medicine; (2017). Accessed December 9, 2023. Available online at:https://kns.cnki.net/kcms2/article/abstract?v=SDjqx_HoHgu-5ymH6E0u08XNB_2wI8HvLxUqsaGh0ILaMPJxYR3VaGtZu7Kdn-rXRZVNHWBc2I9kpPD2XcAlwt7j7TzMjzVcxHLyjb0ffaLMPcovEO9SQCTBVGzTOYBE3v1xhHxG25rY0hoipcVy6Q==&uniplatform=NZKPT&language=CHS

[36] Wei YT Xi ZY Tian SM Zhen YN Xie D. Construction of an indicator system for performance appraisal in operating room nurses of cancer hospitals. Chin J Nurs. (2024) 59:1860–8. doi: 10.3761/j.issn.0254-1769.2024.15.010

[37] Feng M Chen YN. Practical exploration of differential performance appraisal in primary hospitals. Health Econ Res. (2017) 9:37–9. doi: 10.14055/j.cnki.33-1056/f.20170825.010

[38] Wang Y Tang JF Qu G. Hospital operating room operations management: research hotspots and directions for development. Syst Eng Theory Prac. (2018) 38:1778–91. doi: 10.12011/1000-6788(2018)07-1778-14

[39] Ji CX. Effects of the hierarchical management model in operating room nursing. Health Med Res Prac. (2022) 19:110–2. doi: 10.11986/j.issn.1673-873X.2022.04.028

[40] Tanaka MJ Prasad R Miller LA Fleck MM Stiles B Boyne CJ. Team approach: improving Orthopaedic operating room efficiency. JBJS Rev. (2023) 11. doi: 10.2106/JBJS.RVW.23.00036, PMID: 37549236

[41] Zhou GX Fang BY Liu XJ Li GZ. The application effect of flexible work schedule combined with Lead-group nurse management Modein ambulatory Surgeryin ophthalmology department. Chin Health Quality Manage. (2024) 31:46–9. doi: 10.13912/j.cnki.chqm.2024.31.5.11

[42] Luo CX Chang HC Bie FG Xu P Zhang XT Wang Q. Human resource allocation for operating room nursing in general hospitals during the novel coronavirus pneumonia epidemic. Chin Nurs Res. (2020) 34:1126–7. doi: 10.12102/j.issn.1009-6493.2020.07.004

[43] He LJ Shi QL Huang DH Zhang YM. Management practices of mobile nurse pools in the context of a normalized epidemic of novel coronavirus-infected pneumonia. Mod Med J. (2021) 49:1232–4. doi: 10.3969/j.issn.1671-7562.2021.10.020

[44] Griffiths P Saville C Ball J Culliford D Jones J Lambert F. Nursing team composition and mortality following acute hospital admission. JAMA Netw Open. (2024) 7:e2428769. doi: 10.1001/jamanetworkopen.2024.28769, PMID: 39158911 PMC11333978

[45] Beauvais B Pradhan R Ramamonjiarivelo Z Mileski M Shanmugam R. When agency fails: an analysis of the association between hospital agency staffing and quality outcomes. Risk Manag Healthc Policy. (2024) 17:1361–72. doi: 10.2147/RMHP.S459840, PMID: 38803621 PMC11129761

[46] Li A Wang YL Guo Q Qin JX. SWOT analysis of the clinical application of the hourly nurse management model. Guangxi Med J. (2023) 45:333–7. doi: 10.11675/j.issn.0253-4304.2023.03.15

[47] Hassanzadeh H Boyle J Khanna S Biki B Syed F. Daily surgery caseload prediction: towards improving operating theatre efficiency. BMC Med Inform Decis Mak. (2022) 22:151. doi: 10.1186/s12911-022-01893-8, PMID: 35672729 PMC9172609

[48] Schwartz RL Hamlin SK Vozzella GM Randle LN Klahn S Maris GJ. Utilizing telenursing to supplement acute care nursing in an era of workforce shortages. Comput Inform Nurs. (2024) 42:151–7. doi: 10.1097/CIN.0000000000001097, PMID: 38252545 PMC11444343

[49] Ag G Pinto-Carral A Villorejo JS Marqués-Sánchez P. Nurse manager Core competencies: a proposal in the Spanish health system. Int J Environ Res Public Health. (2020) 17:3173. doi: 10.3390/ijerph17093173, PMID: 32370186 PMC7246551

[50] McClelland DC. Testing for competence rather than for “intelligence.”. Am Psychol. (1973) 28:1–14. doi: 10.1037/h0034092 4684069

[51] Niu A Ma H Chen Z Zhu X Luo Y. Exploring the competencies of operating room nurses in mobile surgical teams based on the onion model: a qualitative study. BMC Nurs. (2023) 22:254. doi: 10.1186/s12912-023-01417-3, PMID: 37528375 PMC10394863

[52] Stobinski JX. Perioperative nursing competency. AORN J. (2008) 88:417–36. doi: 10.1016/j.aorn.2008.05.001, PMID: 18790102

[53] Kim C Lee J Lee G. The learning transfer of dementia training program participants: its antecedents and mediating effect on the job competency of geriatric caregivers. Healthcare. (2023) 11:2991. doi: 10.3390/healthcare11222991, PMID: 37998483 PMC10671796

[54] Lun B Yang XY Sun DD Ren LJ Ma S. Effectiveness of in-situ simulation-based training mode for nurse specialists of midwifery. J Nurs. (2023) 30:24–7. doi: 10.16460/j.issn1008-9969.2023.19.024

[55] Lim G Lim AJ Quinn B Carvalho B Zakowski M Lynde GC. Obstetric operating room staffing and operating efficiency using queueing theory. BMC Health Serv Res. (2023) 23:1147. doi: 10.1186/s12913-023-10143-0, PMID: 37875897 PMC10599054

[56] Rekha D Kaliyappan HK. Collaborative robot acting as scrub nurse for cataract surgery (CRASCS). J Robot Surg. (2024) 18:339. doi: 10.1007/s11701-024-02089-0, PMID: 39261441

[57] GuoY WY Huang LH Yin SY. Construction and application of nursing handover information system. Chinese Nurs Manage. (2025) 25:97–100. doi: 10.3969/j.issn.1672-1756.2025.01.019

[58] Zhang XM Yuan XC Li JJ Hu SH Lv XQ. The effect of competence progression stratification training based on Benner’s theory on the comprehensive ability and job competence of nurses in primary hospitals. Chin Nurs Res. (2021) 35:4278–81. doi: 10.12102/j.issn.1009-6493.2021.23.029

[59] Yao YW He GL Zhao TY Li XD Guo Y Li Q. Current working situation of specialist nursing teams in robotic surgery: a national survey of China. Chin J Robo Surg. (2023) 4:48–56. doi: 10.12180/j.issn.2096-7721.2023.01.007

[60] Wang HZ Zhang J Yu Y Zhao Y Li KN Ma HY. Parallel operating rooms: a new model of perioperative nursing process and smart surgical platform management. Pattern Recog Artifici Intell. (2023) 36:867–76. doi: 10.16451/j.cnki.issn1003-6059.202310001

