# Correction: Progress of research on methods of human resource allocation in operating room nursing

**DOI:** 10.3389/fpubh.2025.1641921

**Published:** 2025-09-16

**Authors:** Qiaoling Zhang, Bijun Yu, Yangle Ou, Xiuran Zhou, Shifang Zou, Hui Peng, Xiaoying Yan, Tiemei Shen

**Affiliations:** ^1^Department of Nursing, Guangdong Provincial People's Hospital (Guangdong Academy of Medical Sciences), Southern Medical University, Guangzhou, China; ^2^Department of Nursing, Guangdong Pharmaceutical University, Guangzhou, China

**Keywords:** operating theatre, human resource allocation, nurses, nursing management, manpower allocation methodology

The order of authors in the author list of the published paper was erroneously given as Qiaoling Zhang, Bijun Yu, Yangle Ou, Xiuran Zhou, Shifang Zou, Hui Peng, Tiemei Shen, Xiaoying Yan.

The correct author list reads.

Qiaoling Zhang, Bijun Yu, Yangle Ou, Xiuran Zhou, Shifang Zou, Hui Peng, Xiaoying Yan, Tiemei Shen.

Authors Bijun Yu, Yangle Ou, Xiuran Zhou, Shifang Zou, Hui Peng, Xiaoying Yan, were erroneously assigned to affiliation 1 in the published article instead of affiliation 2. Affiliation 1 has now been removed from these authors and replaced with affiliation 2.

Affiliation 2 was omitted for author Tiemei Shen. This affiliation has now been added.

The original version of this article has been updated.

